# Millimetre Level Accuracy GNSS Positioning with the Blind Adaptive Beamforming Method in Interference Environments

**DOI:** 10.3390/s16111824

**Published:** 2016-10-31

**Authors:** Saeed Daneshmand, Thyagaraja Marathe, Gérard Lachapelle

**Affiliations:** Department of Geomatics Engineering, University of Calgary, PLAN Group Schulich School of Engineering, 2500 University Drive, N.W., Calgary, AB T2N 1N4, Canada; tmarathe@ucalgary.ca (T.M.); gerard.lachapelle@ucalgary.ca (G.L.)

**Keywords:** adaptive GNSS array processing, anti-jammer, blind beamforming, distortionless carrier phase measurements

## Abstract

The use of antenna arrays in Global Navigation Satellite System (GNSS) applications is gaining significant attention due to its superior capability to suppress both narrowband and wideband interference. However, the phase distortions resulting from array processing may limit the applicability of these methods for high precision applications using carrier phase based positioning techniques. This paper studies the phase distortions occurring with the adaptive blind beamforming method in which satellite angle of arrival (AoA) information is not employed in the optimization problem. To cater to non-stationary interference scenarios, the array weights of the adaptive beamformer are continuously updated. The effects of these continuous updates on the tracking parameters of a GNSS receiver are analyzed. The second part of this paper focuses on reducing the phase distortions during the blind beamforming process in order to allow the receiver to perform carrier phase based positioning by applying a constraint on the structure of the array configuration and by compensating the array uncertainties. Limitations of the previous methods are studied and a new method is proposed that keeps the simplicity of the blind beamformer structure and, at the same time, reduces tracking degradations while achieving millimetre level positioning accuracy in interference environments. To verify the applicability of the proposed method and analyze the degradations, array signals corresponding to the GPS L1 band are generated using a combination of hardware and software simulators. Furthermore, the amount of degradation and performance of the proposed method under different conditions are evaluated based on Monte Carlo simulations.

## 1. Introduction

Jamming signals can significantly degrade the performance of receivers or completely deny GNSS position and time services. Many applications primarily relying on GNSS are vulnerable to in-band interference. Even a low-power interference signal can potentially jeopardize GNSS receiver’s performance in a circular region around the source within a radius of several kilometres. Although laws prohibit usage of such devices, low-cost jammers popularly known as personal privacy devices (PPD) can be legally or illegally procured. Spatial processing techniques utilizing an array of antennas can effectively defeat interfering signals regardless of their temporal or spectral characteristics; they surpass time and frequency domain processing methods due to their capability to suppress wideband interference. As long as the cost and power consumption of additional front-ends and antennas are justifiable, spatial processing is one of the most powerful countermeasures against various types of interference and jamming signals.

Beamformers and antenna arrays have been considered effective for a variety of signal processing applications such as radar, wireless communications and speech enhancement. Antenna array processing in GNSS applications has been mostly centered on interference suppression [1,2,3,4]. GNSS array based interference countermeasures can be categorized into two approaches. In the first approach, the beamformer is designed to maximize the radiation pattern in the direction of the satellite signal and minimize the radiation pattern in the direction of the undesired signals as in the minimum variance distortionless response (MVDR) method [5]. Zoltowski [6] draws attention on the usage of this beamformer to suppress interfering signals in GPS applications while avoiding signal attenuation. The optimization is performed for each satellite signal separately and to achieve this, information about the array platform attitude parameters is required. Moreover, the antenna array should be fully calibrated. The second approach for array-based GNSS interference suppression is much simpler in terms of implementation. It totally disregards the desired signals in its optimization and tries to only mitigate the interference signals. This approach is called blind beamforming. It has the drawback of possible loss or attenuation of some satellite signals [6,7,8,9]. However, due to its simplicity, this method is of great interest. Moreover, the array processing functionality can be fully performed before Doppler removal and cross correlation stages of a receiver; hence, this approach can be implemented independently of the receiver structure. GPS Anti-Jam Technology (GAJT^®^) [10] is an example of such an implementation. This product is a single unit GPS anti-jam antenna appropriate for use in military land applications that can be connected to any GPS receiver.

Research on GNSS anti-interference mostly focuses on interference suppression capability and there is less research on the distortion and degradation caused due to the spatial processing itself, particularly for high accuracy applications. Research on interference impact on GNSS receiver parameters has been mostly focused on single antennas [11,12,13]. In the context of array processing, focus has been on the distortion caused by the combination of temporal and spatial processing and when the satellite signal AoA is either estimated or available [1,14,15]. In general, space-time processing i.e., the method that explores both temporal and spatial degrees of freedom, does not provide a linear phase frequency response across the band of interest due to the architecture of the space-time filter. Some efforts to characterize and reduce these distortions are available (e.g., [1,16,17]).

Distortion due to the space-only processing method has comparatively received less attention in the literature. Array-based receivers performing space-only processing may experience phase distortions, which in turn degrades the position and time estimates [18,19,20,21]. Although this can be negligible for applications where high accuracy is not strictly required, these distortions adversely affect carrier phase measurements, thereby not allowing resolution of integer cycle ambiguities. Such phase distortions can be totally compensated in methods such as MVDR; however, this cannot be achieved using blind beamformers since satellite AoA information are not used in beamforming.

This paper focuses specifically on the distortion caused on receiver parameters during blind beamforming. In non-stationary interference scenarios, the array weights should be continuously estimated and updated. Use of a blind beamformer for such scenarios may cause carrier phase degradations that are evaluated in this paper. The degradations are shown for GPS L1 array signals generated using a combination of hardware and software simulators. Moreover, the amount of degradation in different conditions obtained by Monte Carlo simulations is evaluated. A strategy to avoid the distortion on receiver parameters due to the blind beamforming process is also evaluated. Moelker [22] shows that a conventional array structure can cause carrier phase tracking distortions, whereas special array configurations can reduce tracking instability. This approach, however, requires that the array be fully calibrated. Kalyanaraman [23] employs an approach in which the phase distortion is compensated in the carrier phase tracking loop; some modifications are however required in the structure of the GNSS receiver. Although both methods are very effective for MVDR based beamformers, they are not proper choices for blind beamforming because they require initial full calibration and modifications in the receiver structure. These requirements are against the concept of a blind beamformer, i.e., being characterized by its simplicity while providing functionality that is independent of the receiver architecture. As per an approach suggested in [24], the calibration process and receiver modifications are not required. This method takes advantage of the self-coherence characteristics of GNSS signals to first estimate the satellite steering vector and subsequently compensate for the phase differences of the received signal at the antenna array. However, since the steering vector is estimated after interference suppression, only the orthogonal component of the steering vector into the interference subspace can be estimated. This may reduce the steering vector estimation accuracy and consequently cause carrier phase distortions. Additionally, the paper provides a solution for estimating the steering vector of only one satellite signal. The solution for simultaneously estimating the steering vectors of several signals from the covariance matrix before Doppler removal and despreading is not investigated.

Moreover, in all of the methods discussed above, the effect of carrier phase distortion on the receiver position solution has been not evaluated. In this paper, a new method will be devised to keep the simplicity of the blind beamformer structure and at the same time reduce tracking degradations. It is also shown that integer cycle carrier phase ambiguity resolution can be achieved when employing this method in interference environments. This is critical in high precision applications. For example, the base station and the rover receivers in a real time kinematic (RTK) network could be equipped with this beamformer to maintain sub-centimetre level accuracy while suppressing jamming signals. In the scope of this paper, the performance of the proposed blind beamformer is compared against conventional blind beamforming (conventional here means the method that does not consider array symmetry) for carrier phase based positioning.

## 2. Signal Model

Throughout this paper, the following notation is adopted: small bold letters stand for vectors and capital bold letters stand for matrices. Superscripts H, T and * denote conjugate transpose, transpose and conjugate, respectively. vec(A) denotes the vectorization operator obtained by stacking the rows of the matrix A on top of one another. ‖a‖ represents the norm of vector a and ∠a denotes the phase of complex value a. E{} stands for the statistical expectation.

**Figure 1:**
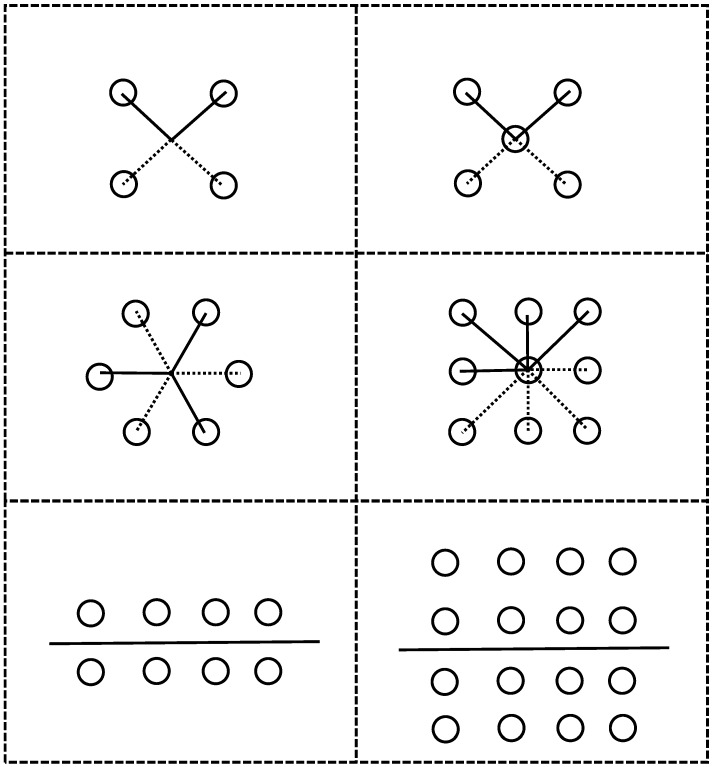
Symmetric array configurations.

Without loss of generality and for the sake of simplicity, only one GNSS signal is considered in the formulations given below. Complex baseband representation of the received signal vector at an arbitrary *N*-element array configuration for the satellite signal and *K* interfering signals can be written as

(1) $$\underset{N\times 1}{x}=\underset{N\times N}{C}\underset{N\times 1}{a}s+\underset{N\times N}{C}\underset{N\times K}{B}\underset{K\times 1}{v}+\underset{N\times 1}{\eta },$$

where B is a matrix whose columns indicate interfering signal steering vectors and η is a complex additive white Gaussian noise vector. s represents the GNSS signal waveform and **v** is a vector specifying *K* interfering signal waveforms. C is an N×N matrix representing constant uncertainties due to any imperfection from antennas to digitizers. The diagonal elements of **C** model the difference in amplitudes and phases due to unequal cable lengths and other electronic parts and its off-diagonal elements represent the coupling coefficients between antennas. In Equation (1), **a** is an N×1 vector representing the steering vector (or array manifold vector) of the satellite signal defined as

(2) $$a=\left[\begin{array}{c} e^{j\frac{2\pi }{\lambda }d^{T}z_{1}}\\ e^{j\frac{2\pi }{\lambda }d^{T}z_{2}}\\ \vdots \\ e^{j\frac{2\pi }{\lambda }d^{T}z_{N}} \end{array}\right],$$

in which λ is the wavelength of the signal and $z_{n}$, *n* = 1, 2, …, *N* is a 3×1 unit vector pointing to the *n*th antenna element and d is a 3×1 unit vector pointing to the satellite direction in the body frame coordinate system. In order to have the beamformer output independent of the gain vector phase changes, the following two assumptions are considered [22]:

1. Symmetric array configuration: For each antenna element (*i*th), there is a corresponding element (*j*th) such that
  (3) $$z_{i}=-z_{j}.$$
  Assuming the body frame coordinate system is located in the centre of the array configuration, the antenna elements are symmetrically located with respect to the origin of the coordinate system. For an odd number of antenna elements, one antenna does not need to follow this rule and it is the one at the centre of the array. Figure 1 shows six possible symmetric circular and rectangular array configurations. For the sake of simplicity in the problem formulation, the array configuration with an even number of antenna elements is employed. Given Equation (3), in a symmetric configuration, the steering vector in Equation (2) can be written as
  (4) $$a=\left[\begin{array}{c} \bar{a}\\ {\bar{a}}^{*} \end{array}\right],$$
  where $\bar{a}$ and ${\bar{a}}^{*}$ are steering vectors corresponding to symmetric sub-arrays.
2. Array weights ($w_{i}$ and $w_{j}$) applied to each symmetric pair of antenna elements (antenna *i* and *j*) are also constrained to be conjugate of each other, such that
  (5) $$w_{i}=w_{j}^{*}$$
  In other words, the array gain vector w can be decomposed as
  (6) $$\underset{N\times 1}{w}=\left[\begin{array}{c} w_{1}\\ w_{2}\\ \vdots \\ w_{N} \end{array}\right]=\left[\begin{array}{c} \underset{\frac{N}{2}\times 1}{w_{U}}\\ \underset{\frac{N}{2}\times 1}{w_{L}} \end{array}\right],$$
  where
  (7) $$w_{U}=w_{L}^{*}.$$

The details on how the above chosen assumptions lead to the distortionless phase response beamformer are given in the next section.

## 3. Proposed Distortionless Phase Response Blind Beamformer

In this section, first a blind beamformer with a distortionless phase response under ideal conditions is studied and then a new method is introduced to keep the phase undistorted under non-ideal conditions while keeping the simple structure of the blind beamformer.

### 3.1. Distortionless Blind Beamforming

**Figure 2:**
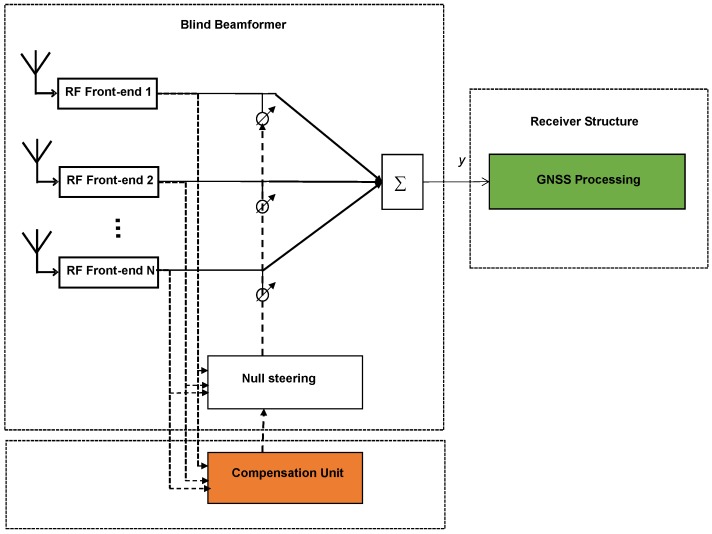
Structure of a blind beamformer and the added compensation unit.

The structure of a blind beamformer is shown in Figure 2. Given Equation (1), the output of the beamformer can be written as

(8) $$y=w^{H}\underset{N\times 1}{x}=w^{H}\underset{N\times N}{C}\underset{N\times 1}{a}s+w^{H}\underset{N\times N}{C}\underset{N\times K}{B}\underset{K\times 1}{v}+w^{H}\underset{N\times 1}{\eta },$$

where w is the array gain vector. As mentioned earlier, a blind beamformer has two drawbacks. Firstly, a potential attenuation for some GNSS signals. Possible loss of one or two satellites in a blind beamformer can be compensated when signals from other constellations are employed, such that there are always enough satellites available to keep the dilution of precision (DOP) at an acceptable level. Secondly, the drawback, which is especially important for high accuracy applications and is the focus of this research, is the degradation due to the tracking phase instability. The array must not deteriorate the performance of tracking, otherwise the positioning performance degrades.

Given the assumptions in Equations (4) and (7) and if it is assumed that the array is fully calibrated, then C can be compensated and considered as an identity matrix in Equation (8) and the following relation always holds:

(9) $$∠(w^{H}as)=∠s,$$

where ∠s is the phase of the signal. Therefore, the phase values of the GNSS signals become independent of the weight coefficients. This avoids phase fluctuations while updating the weights and leads to stable tracking parameters. When the uncertainty matrix C is considered, this is not valid anymore and C should be known a priori or estimated. In the next section, by taking advantage of the specific structure of the array configuration mentioned in Equation (4) and the gain coefficients constraint mentioned in Equation (7), a real-time compensation approach for unknown C is proposed.

### 3.2. Proposed Phase Compensation Approach

In order to estimate C without errors, a full calibration procedure is required. It is traditionally conducted in an anechoic radio frequency (RF) chamber such that the antenna array receives a signal from a precisely known direction. GNSS array calibration can also be carried out with actual line of sight signals since the satellite positions are known [25,26]. Moreover, for the purpose of calibration, array attitude parameters are required, which can be derived using either accurate measurements or IMUs.

In this section, by taking advantage of the special array configuration, a phase compensation approach is proposed to compensate for beamforming phase changes. For this approach, the array needs not be fully calibrated and knowledge of array platform attitude parameters and the direction of the incident signals are not required. Moreover, the following estimation process is independent of the receiver structure. The compensation unit can be implemented within the structure of the conventional blind beamformer without feedback from the GNSS receiver (as shown in Figure 2). The process is explained as follows.

Considering Equations (8) and (9), a compensation matrix is applied to the received signal vector to satisfy the following condition:

(10) $$∠(w^{H}\bar{C}\bar{x})=0,$$

where $\bar{C}$ is the compensating matrix. Herein, it is also assumed that the calibration signal $s_{cal}$ with a high SNR is known and tracked by the beamformer during the estimation process. In Equation (10), $\bar{x}$ denotes the received signal vector x in which the phase of the calibration signal is kept constant during the estimation process. Therefore, $∠(w^{H}\bar{C}\bar{x})=∠w^{H}\bar{C}Ca+∠s_{cal}$ (for simplicity and better illustration, $∠s_{cal}$ in the rest of the analysis and later in Monte Carlo simulations is assumed zero). As long as $\bar{C}C$ is a block conjugate centrosymmetric matrix, the array configuration is symmetric (Equation (4)) and the array gain vector is conjugate symmetric (Equation (7)), $∠w^{H}\bar{C}Ca=0$. During the normal operation of the beamformer, these assumptions guarantee that only the phase distortion due to the array imperfection is compensated and the phase values of satellite signals used for carrier phase based positioning remain intact.

In order to estimate $\bar{C}$, assume this matrix is decomposed as left, right, upper and lower block matrices and $\bar{x}$ is decomposed as upper and lower vectors as follows:

(11) $$\bar{C}=\left[\begin{array}{cc} \underset{\frac{N}{2}\times \frac{N}{2}}{{\bar{C}}_{LU}} & \underset{\frac{N}{2}\times \frac{N}{2}}{{\bar{C}}_{RU}}\\ \underset{\frac{N}{2}\times \frac{N}{2}}{{\bar{C}}_{LL}} & \underset{\frac{N}{2}\times \frac{N}{2}}{{\bar{C}}_{RL}} \end{array}\right],\;\bar{x}=\left[\begin{array}{l} \underset{\frac{N}{2}\times 1}{x_{U}}\\ \underset{\frac{N}{2}\times 1}{x_{L}} \end{array}\right].$$

It is easy to conclude that the relation in Equation (10) holds if

(12) $${\left({\bar{C}}_{LU}x_{U}+{\bar{C}}_{RU}x_{L}\right)}^{*}=\left({\bar{C}}_{LL}x_{U}+{\bar{C}}_{RL}x_{L}\right).$$

Matrices and vectors in Equation (12) can be rearranged as

(13) $${\left(X_{U}c_{LU}+X_{L}c_{RU}\right)}^{*}=X_{U}c_{LL}+X_{L}c_{RL},$$

in which

(14) $$\underset{\frac{N}{2}\times \frac{N^{2}}{4}}{X_{L}}=\left[\begin{array}{cccc} x_{L}^{T} & 0_{1\times \frac{N}{2}} & \cdots & 0_{1\times \frac{N}{2}}\\ 0_{1\times \frac{N}{2}} & x_{L}^{T} & \cdots & 0_{1\times \frac{N}{2}}\\ \vdots & \vdots & \ddots & \vdots \\ 0_{1\times \frac{N}{2}} & 0_{1\times \frac{N}{2}} & \cdots & x_{L}^{T} \end{array}\right]\;\underset{\frac{N}{2}\times \frac{N^{2}}{4}}{X_{U}}=\left[\begin{array}{cccc} x_{U}^{T} & 0_{1\times \frac{N}{2}} & \cdots & 0_{1\times \frac{N}{2}}\\ 0_{1\times \frac{N}{2}} & x_{U}^{T} & \cdots & 0_{1\times \frac{N}{2}}\\ \vdots & \vdots & \ddots & \vdots \\ 0_{1\times \frac{N}{2}} & 0_{1\times \frac{N}{2}} & \cdots & x_{U}^{T} \end{array}\right]\;\begin{array}{c} \begin{array}{l} \underset{\frac{N^{2}}{4}\times 1}{c_{LU}}=vec({\bar{C}}_{LU}^{*})\\ \underset{\frac{N^{2}}{4}\times 1}{c_{RU}}=vec({\bar{C}}_{RU}^{*})\\ \underset{\frac{N^{2}}{4}\times 1}{c_{LL}}=vec({\bar{C}}_{LL}) \end{array}\\ \underset{\frac{N^{2}}{4}\times 1}{c_{RL}}=vec({\bar{C}}_{RL}) \end{array}.$$

Equation (13) can be written as

(15) $$\overset{X}{\overset{︷}{\left[\begin{array}{cccc} X_{U}^{*} & X_{L}^{*} & -X_{L} & -X_{U} \end{array}\right]}}\overset{c}{\overset{︷}{\left[\begin{array}{c} c_{LU}\\ c_{RU}\\ c_{RL}\\ c_{LL} \end{array}\right]}}=\underset{\frac{N}{2}\times N^{2}}{X}\underset{N^{2}\times 1}{c}=\underset{N^{2}\times 1}{0},$$

in which c is of interest to estimate and consists of elements of the compensation matrix in a vector form. In order to estimate c, ‖Xc‖ should be minimized. Hence c can be estimated as

(16) $$\underset{\left\|c\right\|=1}{\min}\;c^{H}X^{H}Xc.$$

c cannot be determined uniquely from this optimization by performing singular value decomposition (SVD) since $X^{H}X$ is rank deficient. In fact, c belongs to the null space of $X^{H}X$ with the dimension of $\left(N^{2}-N/2\right)$ and is equivalent to the Eigen vector corresponding to the smallest Eigen value of $X^{H}X$. In order to increase the rank, more uncorrelated data samples should be employed as $E\left\{X^{H}X\right\}$ instead of $X^{H}X$ in the minimization in Equation (16); this in the above problem means that the array should receive the signal from different directions. It should also be noted that $\bar{C}$ is not necessarily equal to the calibration matrix ($C^{-1}$). In fact, as long as $\bar{C}C$ is a block conjugate centrosymmetric matrix, the condition in Equation (10) holds.

The compensation unit is independent of the null steering and can be considered as a parallel unit in the pre-despreading beamformer. It should however be noted that compared to the conventional blind beamformer, the degree of freedom of the array is reduced by half by employing this approach. This is a trade off between the number of interfering signals that can be suppressed and the accuracy of the position estimates obtained after mitigation.

### 3.3. Interference Nullifying

In the last step, the array gain vector to nullify interference can be obtained based on the estimated compensation matrix in a constrained optimization. Minimizing the output of the beamformer leads to the following optimization:

(17) $$\begin{array}{l} \min{\left\|y\right\|}^{2}=\min\;w^{H}\bar{C}E\left\{xx^{H}\right\}{\bar{C}}^{H}w\\ e^{H}w=1,\;w_{U}=w_{L}^{*}, \end{array}$$

where

(18) $$e={\left[\begin{array}{cccc} 1 & 0 & \cdots & 0 \end{array}\right]}^{T}.$$

The first constraint avoids the trivial solution which is w=0 and the second guarantees that the symmetric gain vector satisfies the relation in Equation (10). In order to solve the optimization problem, given Equations (8) and (11), the beamformer output is written as

(19) $$y=w_{U}^{H}{\bar{C}}_{LU}x_{U}+w_{L}^{H}{\bar{C}}_{LL}x_{U}+w_{U}^{H}{\bar{C}}_{RU}x_{L}+w_{L}^{H}{\bar{C}}_{RL}x_{L},$$

and hence

(20) $$\begin{array}{l} {\left\|y\right\|}^{2}=w_{U}^{H}\left({\bar{C}}_{LU}x_{U}+{\bar{C}}_{RU}x_{L}\right){\left({\bar{C}}_{LU}x_{U}+{\bar{C}}_{RU}x_{L}\right)}^{H}w_{U}+w_{L}^{H}\left({\bar{C}}_{LL}x_{U}+{\bar{C}}_{RL}x_{L}\right){\left({\bar{C}}_{LL}x_{U}+{\bar{C}}_{RL}x_{L}\right)}^{H}w_{L}\\ +w_{U}^{H}\left({\bar{C}}_{LU}x_{U}+{\bar{C}}_{RU}x_{L}\right){\left({\bar{C}}_{LL}x_{U}+{\bar{C}}_{RL}x_{L}\right)}^{H}w_{L}+w_{L}^{H}\left({\bar{C}}_{LL}x_{U}+{\bar{C}}_{RL}x_{L}\right){\left({\bar{C}}_{LU}x_{U}+{\bar{C}}_{RU}x_{L}\right)}^{H}w_{U}. \end{array}$$

Considering the symmetry in the array, and Equations (7) and (12), it can be easily concluded that $w_{U}^{H}$ estimated from the following minimization also minimizes the output of the beamformer in Equations (17) and (19):

(21) $$\begin{array}{l} \min\;w_{U}^{H}E\left\{\left({\bar{C}}_{LU}x_{U}+{\bar{C}}_{RU}x_{L}\right){\left({\bar{C}}_{LU}x_{U}+{\bar{C}}_{RU}x_{L}\right)}^{H}\right\}w_{U}\\ e^{H}w=1. \end{array}$$

The solution for this minimization is straightforward by using a Lagrange multiplier approach. Another approach is directly solving the optimization in Equation (17) similar to the one performed in [21]. In the next section, the performance of the compensation approach is evaluated by running Monte Carlo simulations.

## 4. Monte Carlo Simulations

Incorporating the simulation of perturbations in array element positions or modifying the array miscalibration errors and generating GNSS signals with different signal settings is a time consuming task. Therefore, a Monte Carlo approach, which is an alternative and comparatively simpler way to evaluate the performance of the proposed method in different scenarios, is presented in this section; in the next section the effectiveness of the approach in a receiver and position domain is verified for one set of GPS array signals. The parameter settings used in Monte Carlo approach are given in Table 1. Three different simulation parameter settings (Case 1, 2 and 3) are considered to cater to the tests described in this section. The various parameters being considered in Table 1 includes the parameters that determine the antenna characteristics and setting for simulations. Antenna element position perturbation models the possible deviations in the measured antenna positions. The number of iterations indicates the number of simulations performed repeatedly with each case of parameter settings. The field in the table named “Number of samples in simulation” signifies the number of random spatially distributed samples of a signal used for estimating the compensation matrix. A continuous wave signal with an SNR of 12 dB is considered for the signal used for estimating the compensation matrix. Independent samples representing the GNSS signals arriving from different directions are generated to evaluate the phase distortion after applying the compensation matrix. The SNR of 15 dB is chosen for these samples.

Unequal cable lengths, mutual coupling among array elements, antenna phase and gain mismatches, and phase centre variations can affect the resulting phase and amplitude of the received signal at each antenna. In [26], it was shown that the calibration uncertainties can be modeled as a constant unknown matrix multiplied by an AoA-dependent perturbation matrix. The amount of this perturbation depends on the array design and the type of antenna being employed. It can be insignificant or has the major contribution in the overall uncertainties. For precise and high accuracy applications, ideally, the antenna should receive only signals above the horizon and reject all signals below the user horizon plane of the antenna and have a known and stable phase centre that is co-located with the geometrical centre of the antenna. Generally, for GNSS antennas, the AoA dependency of incident signals is modeled by phase centre stability.

The antenna gain patterns considered in the simulations correspond to the Novatel GPS Antenna 501 [27]. In Case 2, the simulation model considers a phase centre stability ranging from 0 to 5 mm. A constant term of the calibration uncertainties is randomly generated as a normalized complex-value matrix in each run. Off-diagonal elements of this matrix represent the coupling between antenna elements and are chosen to be between 0 and 0.5.

As discussed, in addition to the array gain, there is an induced phase due to the beamforming process that should be avoided in order to have undistorted phase from a beamformer. In the following simulations, the amount of this induced phase is evaluated. Additionally, for the sake of simplicity in illustrations, only induced phase due to the beamformer is considered and the phase of satellite signal is assumed zero. Therefore, any phase distortion appears in the form of a non-zero phase and it consists of an imaginary part.

**Figure 3:**
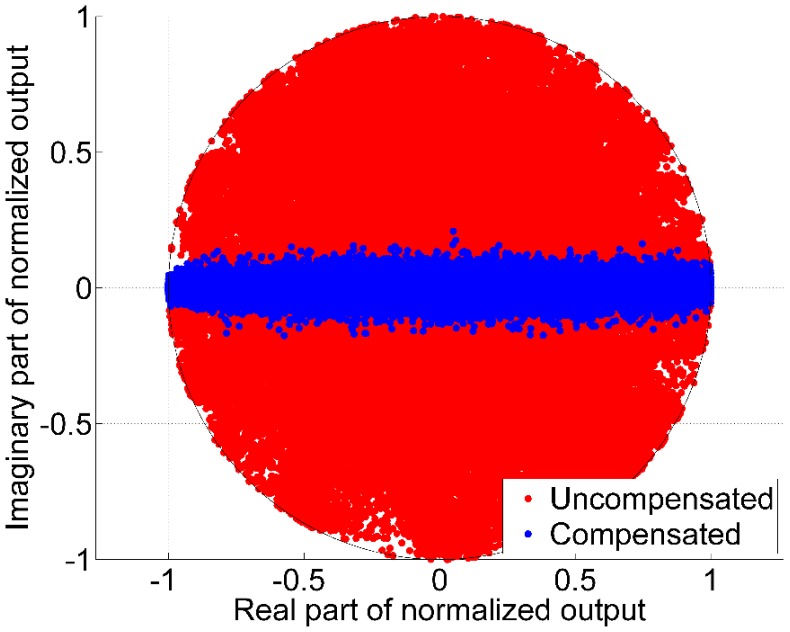
Comparison of uncompensated and compensated method—scatter plot of phase distortion.

In the first simulation, the induced phase is illustrated (Figure 3) in real-imaginary component plane for the conventional and proposed beamformers. On performing conventional blind beamforming, there is no constraint in terms of phase variations; therefore, both real and imaginary components are present. In the proposed method, using the symmetric nature of the array and applying the compensation matrix, the imaginary part is suppressed as described in Equation (10). This behaviour in the output is illustrated in Figure 3. The simulations were performed using the settings for Case 1 listed in Table 1. In each run, the array gain vector and calibration uncertainty matrix are randomly chosen (matrix C and vector w in Equation (8)). As seen from Figure 3, in the conventional uncompensated beamformer all combinations of real and imaginary components of the output are present and therefore a phase distortion and jumps are expected while updating the beamformer gain vector. To the contrary, upon use of the proposed method, imaginary components spread only around a narrow band and provide phase constant beamformer output. This illustration qualitatively indicates the potential benefits of the proposed method. To quantify the enhancements offered by the proposed method, a ratio metric is used. The Ratio Metric (RM), expressed in dB, is defined as follows:

(22) $$RM=10\log_{10}\left(\frac{\sigma_{R}^{2}}{\sigma_{I}^{2}}\right),$$

where $\sigma_{R}^{2}$ is the variance of the real part of the output and $\sigma_{I}^{2}$ is the variance of the imaginary part of the beamformer output. The method is further assessed for different levels of antenna position perturbations and for different number of antenna array elements. The results corresponding to these experiments are analyzed in the following subsections.

### 4.1. Effects Due to Antenna Element Position Perturbations

**Figure 4:**
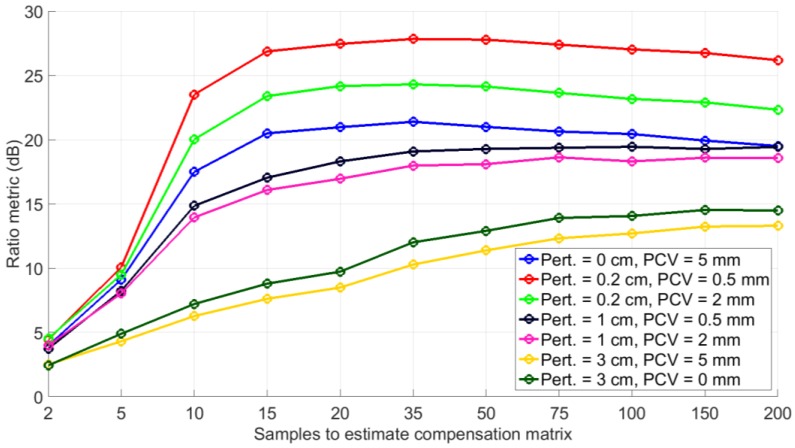
Ratio metric comparison for different element position perturbations and phase centre variations.

Generally, even though the array configuration is chosen in advance, the positions of the antenna array element’s phase centres are not precisely known due to physical perturbation, phase centre variations and other uncertainties. This gives rise to antenna element position perturbations. These perturbations lead to deviation from the symmetric assumption in the array configuration. For the purpose of this analysis, perturbations in the range of 0 to 3 cm are considered. Perturbations of the chosen magnitude were added to each direction/co-ordinate (i.e., X, Y and Z). The Monte Carlo simulations for an eight element symmetric array were done using the settings provided in Table 1 (Case 2). A comparison of the ratio metrics for different antenna element perturbations is shown in Figure 4.

A higher value of the ratio metric indicates that the output has higher contributions from the real values compared to the imaginary values. From Figure 4 it can be seen that with increasing perturbations, the ratio metric decreases and consequently the beamformer performance degrades. However, the curves in the Figure 4 also indicate that, even for larger perturbations, though one does not expect this in actual arrays, ratios with considerable magnitude are maintained between real and imaginary components. This indicates that the proposed approach is not susceptible to array perturbations. In Figure 4, it can also be observed that by considering more data samples to form the correlation matrix in Equation (16), the rank of $E\{X^{H}X\}$ is increased; consequently, better estimation of c is possible, as discussed in Section 3.

### 4.2. Effects of the Number of Antenna Elements

**Figure 5:**
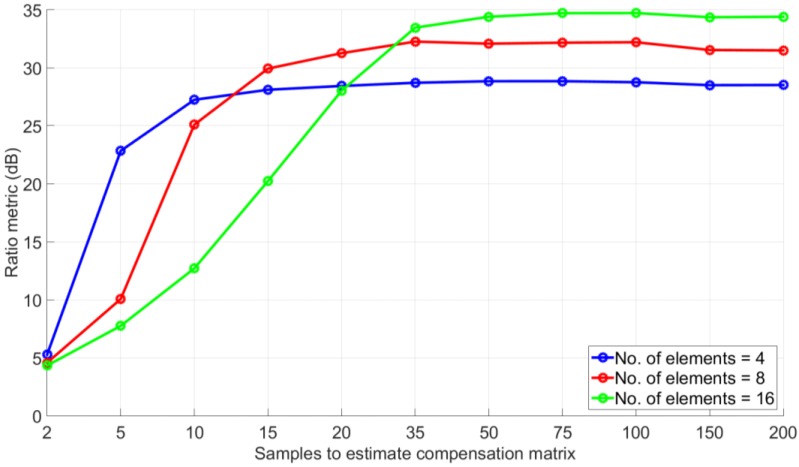
Ratio metric comparison for different number of antenna elements.

Performance of any beamforming differs for different numbers of antenna array elements. The performance of the method is now evaluated for different number of elements; however, a symmetric configuration is maintained as shown in Figure 1. The comparison of the ratio metrics for different numbers of elements as a function of the number of samples used to estimate the compensation matrix is shown in Figure 5. The settings used for Monte Carlo simulations are as given in Table 1 (Case 3). For a smaller number of antenna element, the steady state ratio metric was achieved with a lower number of samples used to estimate the compensation matrix while for a higher number of antenna elements (e.g., sixteen elements), the steady state was reached with a higher number of samples as compared to the lower number of antenna elements (e.g., with four elements). However, the ratio metric is larger for the array with the greater number of antenna elements. Therefore, as expected, there is a trade off between the required samples to estimate the compensation matrix and performance.

## 5. Carrier Phase Distortion Analysis

Herein, GNSS array data are simulated using the recorded GPS digital baseband signals generated with a hardware simulator. Precise array signals are simulated using the simulation method outlined in [28]. The digital baseband samples are collected using a Spirent GSS 7700 simulator. The GSS 7700 is a multiple GPS satellite signal simulator that allows one to choose the user position and the user dynamics profile. The carrier Doppler, code delays and the navigation data bits extracted by processing collected intermediate frequency (IF) samples are used to generate array signals by employing the method of phase translations. The user position and the array configuration can be selected by the user. Precise steering vectors are available for signal generation, as exact locations of the satellites are known a priori. A GPS signal scenario corresponding to a static user is considered for simulations; however, the signal variations due to the satellite motion are appropriately accounted. The pseudo random number (PRN), azimuth and elevation of the simulated satellites are given in Table 2. An antenna array with five antenna elements with symmetric configuration as shown in Figure 1 is used for the analysis. Due to frequency regulations, outdoor RF power transmission in the GNSS frequency bands is prohibited. In order to test and evaluate the performance of the method described earlier under actual interference signals, these are generated in software and added to the recorded samples. One CW interfering signal in the GPS L1 frequency band is considered and, in order to model the changing interference environment, it is repeatedly turned on and off. This requires the beamformer to update its array gain vector regularly. Interference parameters are given in Table 3.

**Figure 6:**
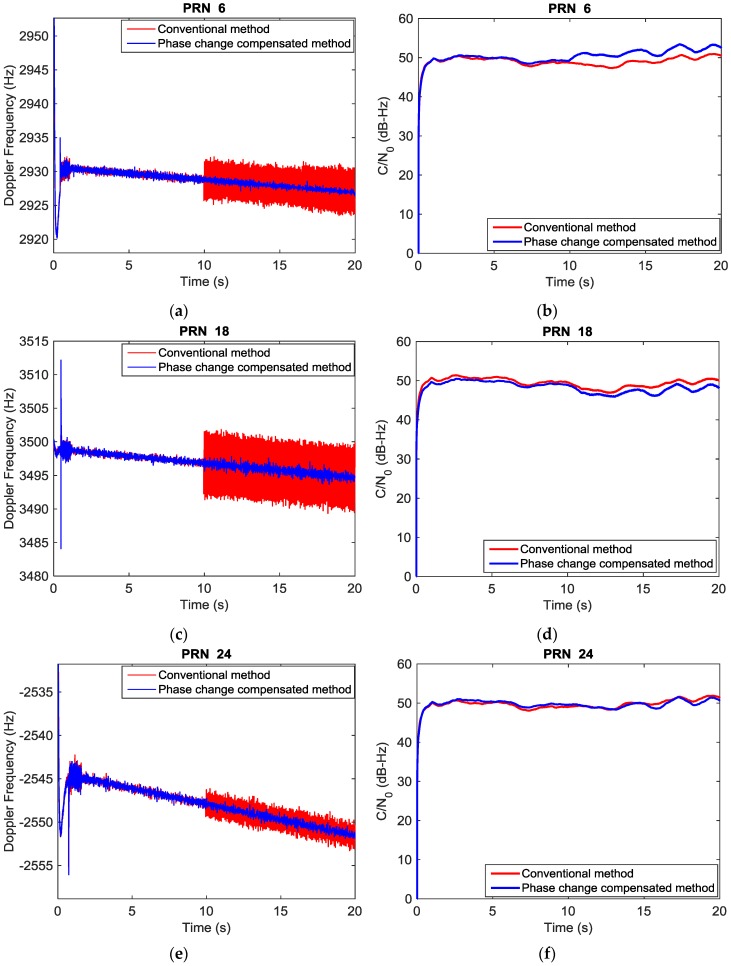
Doppler frequency and C/N_0_ comparison between two blind beamforming approaches. (**a**) Doppler Frequency PRN 6; (**b**) C/N_0_ PRN 6; (**c**) Doppler Frequency PRN 18; (**d**) C/N_0_ PRN 18; (**e**) Doppler Frequency PRN 24; (**f**) C/N_0_ PRN 24.

The analysis was conducted using a multi-antenna GNSS software receiver that was developed starting from the single antenna GSNRx software receiver [29]. The generated array data are processed and the pseudorange and carrier phase measurements are obtained using this multi-antenna receiver after applying blind beamforming methods. Doppler frequency and C/N_0_ for the conventional (phase uncompensated) method and the new approach (phase compensated) are compared. In the conventional beamformer, there is no assumption on the array configuration or having symmetric conjugate gain vector. Moreover, the compensation matrix is not applied to the received signal vector. Results for three PRNs are shown in Figure 6. Interference is present 10 s after running the process in the conventional approach, as expected, the carrier phase turns unstable due to the array gain updates, which in turn causes noisier carrier phase measurements. The phase degradations observed in the plots are not due to unintentional nulls in the direction of the satellite signals in the blind beamformer since the C/N_0_ values show no attenuations. As observed in Figure 6, the C/N_0_ values for both approaches are high and almost the same.

Some preliminary experiments analyzing the accuracy of code phase based positioning indicated marginal improvements in some cases and significant improvements in others, depending on the interference scenario. To the contrary, significant improvements were always seen in the position results derived using the carrier phase measurements, irrespective of the interference scenario. Further analyses on the code phase measurements are required to assess the improvements obtained from the proposed method. The scope of this paper is restricted to carrier phase based positioning.

RTKLIB [30] is used to compare the position results between compensated and uncompensated blind beamformers. Similar to other such software, RTKLIB uses initially a combination of pseudorange and carrier phase measurements. As mentioned previously, the pseudorange and carrier phase measurements are generated by the GSNRx software receiver after applying blind beamformer weights. These measurements are converted to a format, namely RINEX, compatible with the RTKLIB. Using this combination of measurements, RTKLIB attempts to resolve the carrier phase ambiguities as integer values. If this is achieved, the ambiguities become deterministic and are removed from the state vector; otherwise, they remain stochastic and their values are updated in the state vector at each measurement epoch as numbers with fractions, referred to as a float solution.

**Figure 7:**
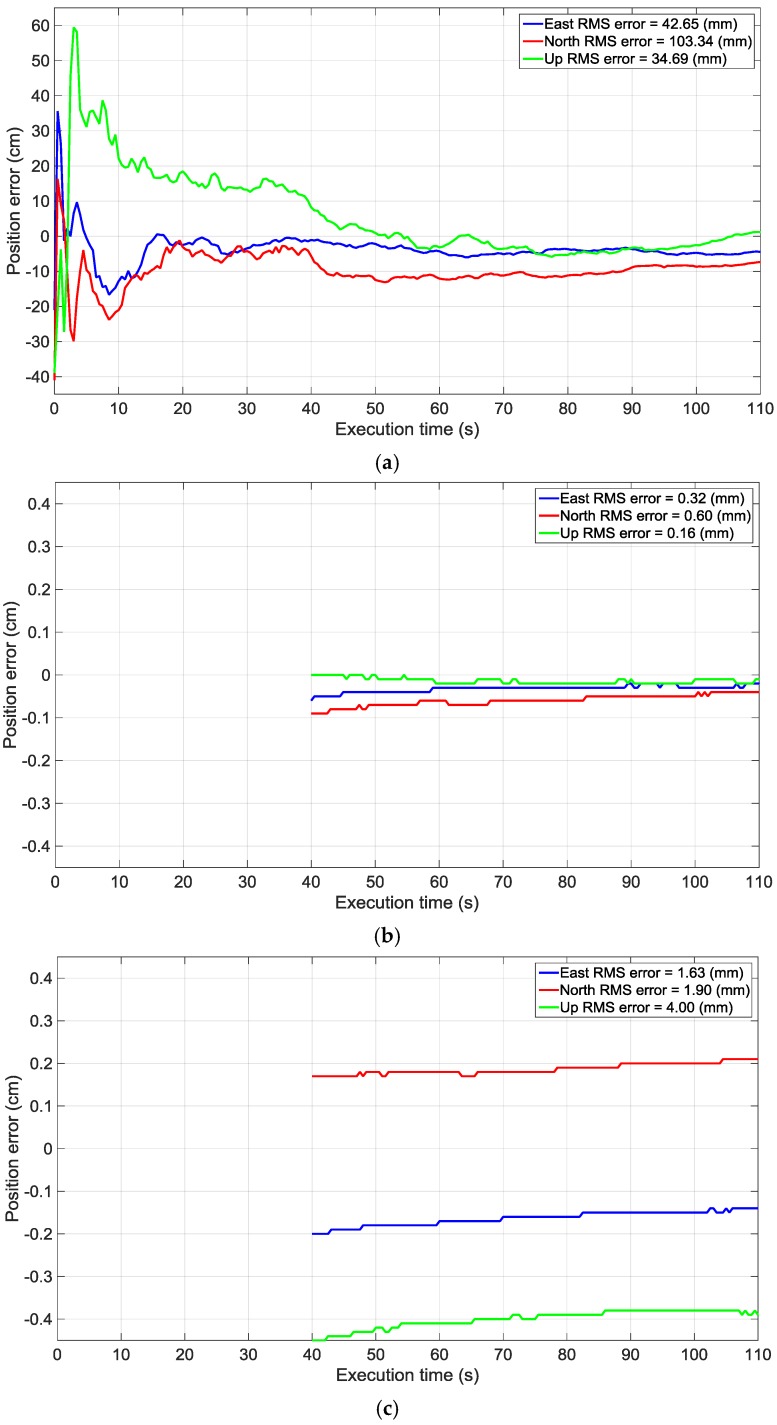
Position errors for: (**a**) Uncompensated blind beamformer (float ambiguity solution); (**b**) Compensated blind beamformer (fixed solution); and (**c**) Compensated blind beamformer (fixed integer ambiguity solution) considering the multipath and non-ideal antennas.

Figure 7 shows the errors in the ENU coordinate system (East, North and Up) for compensated and uncompensated beamformers. The errors for the compensated beamformer are shown after achieving a fixed integer carrier phase ambiguity position solution. For both cases, the same PRNs are employed in the position solutions and therefore the DOP was the same for both solutions. Therefore, the only difference is due to the carrier phase degradation, which was compensated in the proposed method. In the conventional blind beamformer approach, the virtual array phase centre is a function of signal AoA, which is different for different satellite signals. This prevents the receiver from achieving a fixed position solution based on carrier phase measurements whereas in the proposed method the array phase differences for each satellite signal are compensated. Therefore the virtual phase centre of the array becomes independent of incident signals’ AoA and is at the centre of the array configuration. As observed, the proposed blind beamformer is able to mitigate the interference and at the same time allow the receiver to achieve a carrier phase integer ambiguity position solution with millimetre-level accuracy. For the data set analyzed herein, a NovAtel ProPak V3 was used as the reference receiver and the original signal generated by the hardware simulator was processed with this receiver to model a zero baseline scenario.

The RMS values of errors in 3D positioning are 117 mm and 0.7 mm for uncompensated and compensated beamformers respectively after convergence, which shows a significant improvement in the compensated beamformer.

However, this level of accuracy is achieved by neglecting the error contributions from carrier phase multipath, non-ideal antennas and other residual differential errors. In order to assess this effect on accuracy, another test is performed by adding carrier phase multipath and antenna array imperfections into the simulated data. Residual errors due to non-ideal antenna elements, coupling and other imperfections after estimating the compensation matrix are modeled based on the analysis in Section 3 and added to the carrier phase measurements. Herein, the order of the errors is obtained from a Monte Carlo simulation with the following settings: phase instability = 5 mm, SNR of calibrating signal = 10 dB, number of samples for estimating compensation matrix = 200, perturbation error = 3 cm). The phase instability (5 mm) and the antenna gain patterns are chosen based on the typical values given in the datasheet of the Novatel GPS Antenna 501 [27].

**Figure 8:**
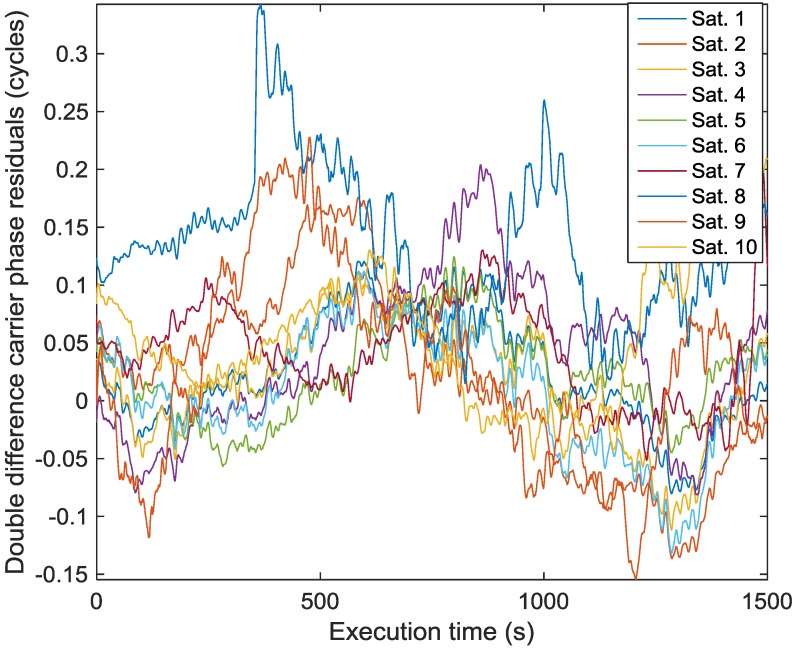
Double difference carrier phase multipath used in simulation.

Since the proposed method does not bring in any phase distortion due to the spatial processing to any of the incident signals including reflected signals, the carrier phase multipath error model stays unaltered. Therefore, in order to simulate close to actual signal conditions, a modeled carrier phase multipath is added to the error-free carrier phase measurements. The procedure adopted to obtain the carrier phase multipath model is as follows. A differential static GPS survey was conducted over a distance of 20 km. Two NovAtel Propak-V3 receivers and geodetic antennas were used to collect data 20 times per second. The carrier phase ambiguities were resolved as integer values and a least-squares method (to avoid smoothing effects) was used to generate the double difference carrier phase residuals, which include noise, differential atmospheric effects and multipath. High frequency thermal noise is at the sub-millimetre level but was nevertheless removed using a low pass filter. The differential atmospheric effect is expected to be at the millimetre level at the most. The remaining residuals constitute a valid measure of carrier phase multipath and are shown in Figure 8.

These multipath effects and antenna array imperfections were added to the phase measurements obtained in the receiver after beamforming. Figure 7 also shows the errors in the ENU coordinates for the proposed method under the modeled carrier phase multipath and the antenna array imperfections. Although for this data set the beamformer can still achieve carrier phase integer ambiguity resolution, the level of accuracy decreases slightly. The 3D position RMS error value becomes 4.7 mm, instead of 0.7 mm in the absence of multipath and non-ideal antennas, as described earlier.

The proposed beamformer functions are independent of the architecture of the receiver being used. Therefore, any multipath mitigation method can be added to the output of the proposed beamformer. Additionally, when the receiver is equipped with an antenna array, spatial domain processing methods could be used to alleviate these errors, which have not been investigated in this paper.

## 6. Conclusions

This paper investigated the phase distortions in adaptive blind beamforming. The results showed that, although the conventional blind beamformer can effectively suppress jammers or interference, it is not immune to carrier phase distortion.

Herein, a solution for the above problem was proposed. The proposed approach took advantage of the symmetry in the chosen array configuration, phase compensation, and conjugate symmetric array gain vector constrained optimization. Contrary to similar work, it neither requires the array to be fully calibrated nor imposes any modifications on a GNSS receiver architecture.

The experiment, which was performed using the sample data generated from the combination of hardware and software simulators, showed that the new method resulted in position estimates at millimetre-level accuracy for the blind beamforming method in the interference scenario and at the few millimetres level when multipath and non-ideal antennas are considered. Moreover, errors on the position solution were compared between the new approach and the conventional blind beamformer using a multi-antenna software receiver developed during the course of this research. For comprehensive evaluation, Monte Carlo simulations were performed to analyze performance of the proposed method in different conditions.

## Figures and Tables

**Table 1:** Monte Carlo simulation parameters.

Parameter	Setting		
	Case 1	Case 2	Case 3
Perturbation in position of array element (in cm) and Phase centre stability or phase centre variation (PCV) (in mm)	(0.1, 0.0)	(0.0, 5.0); (0.2, 0.5); (0.2, 2.0); (1.0, 0.5); (1.0, 2.0); (3.0, 5.0); (3.0, 0.0)	(0.1, 0.0)
Number of iterations	100	1000	1000
Number of antenna elements (array configurations are shown in Figure 1)	8	8	4, 8, 16
Number of samples in simulation	1000	2, 5, 10, 15, 20, 35, 50, 75, 100, 150, 200	2, 5, 10, 15, 20, 35, 50, 75, 100, 150, 200

**Table 2:** Satellite visibility corresponding to the simulated data.

Satellite PRN	Azimuth (°)	Elevation (°)
3	283	18
6	281	32
10	35	14
16	282	62
18	150	22
21	128	79
24	58	40
25	330	11

**Table 3:** Interference scenario.

Interference	Parameters Description
Single CW source	Azimuth = 40° Elevation = 10° The interference-to-noise density ratio = 15 dB Frequency offset with respect to the L1 centre frequency = 1000 Hz
